# Rational design and validation of an anti-protein kinase C active-state specific antibody based on conformational changes

**DOI:** 10.1038/srep22114

**Published:** 2016-02-25

**Authors:** Darlene Aparecida Pena, Victor Piana de Andrade, Gabriela Ávila Fernandes Silva, José Ivanildo Neves, Paulo Sergio Lopes de Oliveira, Maria Julia Manso Alves, Lakshmi A. Devi, Deborah Schechtman

**Affiliations:** 1 Departamento de Bioquímica, Instituto de Química, Universidade de São Paulo, SP, Brazil; 2 Departamento de Patologia, A.C. Camargo Cancer Center, São Paulo, SP, Brazil; 3 Laboratório Nacional de Biociências, Centro Nacional de Pesquisa em Energia e Materiais, Campinas, SP, Brazil; 4 Department of Pharmacology and Systems Therapeutics, Icahn School of Medicine at Mount Sinai, New York, NY, USA

## Abstract

Protein kinase C (PKC) plays a regulatory role in key pathways in cancer. However, since phosphorylation is a step for classical PKC (cPKC) maturation and does not correlate with activation, there is a lack of tools to detect active PKC in tissue samples. Here, a structure-based rational approach was used to select a peptide to generate an antibody that distinguishes active from inactive cPKC. A peptide conserved in all cPKCs, C2Cat, was chosen since modeling studies based on a crystal structure of PKCβ showed that it is localized at the interface between the C2 and catalytic domains of cPKCs in an inactive kinase. Anti-C2Cat recognizes active cPKCs at least two-fold better than inactive kinase in ELISA and immunoprecipitation assays, and detects the temporal dynamics of cPKC activation upon receptor or phorbol stimulation. Furthermore, the antibody is able to detect active PKC in human tissue. Higher levels of active cPKC were observed in the more aggressive triple negative breast cancer tumors as compared to the less aggressive estrogen receptor positive tumors. Thus, this antibody represents a reliable, hitherto unavailable and a valuable tool to study PKC activation in cells and tissues. Similar structure-based rational design strategies can be broadly applied to obtain active-state specific antibodies for other signal transduction molecules.

The Protein kinase C (PKC) family of serine-threonine kinases is composed of at least ten isoenzymes, that play significant regulatory roles in key pathways in cancer such as, cell motility, survival, cell cycle and differentiation. Recent studies have demonstrated that mutations in PKC usually lead to a loss of function suggesting a tumor suppressor role for PKCs. In some tumors, such as breast cancer, PKC mutations are rare^1^ and there are difficulties to establish the cause-effect of the PKC family of proteins. This may be due to complex biological activities involving the different isoenzymes coupled with difficulties in measuring expression and activation status of each isoenzyme^2^. Furthermore, there is generally a poor correlation between mRNA levels and protein expression levels, due to antibody specificity or data for mRNA levels generated from whole tissues instead of microdissected tumor cells. Since phosphorylation is a step for PKC protein folding (maturation) more than a marker of protein activation as known for Erk, Akt or JNK, we currently can only detect PKC isoenzyme activation status or substrate phosphorylation in cell cultures but not in tumor tissue^2^.

Difficulties to determine PKC expression and activation status are partly due to the fact that the PKC family is composed of at least ten different isoenzymes divided into three sub-families according to their activation requirements. Classical (cPKCs): α, βΙ, βΙΙ and γ are calcium dependent and activated by phosphatidyl serine and diacylglycerol , novel (nPKCs): are calcium independent, but depend on the same lipids as cPKCs for their activation and atypical (aPKCs): λ and ζ are calcium independent and activated by different lipids such as ceramide^3^. PKC activation requires translocation of the enzyme to membranes^4^ where it modulates signaling cascades through phosphorylation of serine and threonine amino acid residues of a large selection of proteins.

Most antibodies that are thought to detect active kinases are directed against phosphorylation sites considered essential for kinase activation such as the activation loop^5^. These antibodies have been used in a variety of assays to determine cancer prognosis with limited success since tissue processing conditions may lead to loss of phosphate groups and false negative signals^6^. Further, in the case of cPKCs, phosphorylation does not correlate with activation^7^,^8^,^9^. PKCs undergo three phosphorylations at specific sites in the catalytic domain activation loop, turn motif, and hydrophobic motif. These phosphorylations confer stability, catalytic competence and proper subcellular localization of active kinase^9^. In cPKCs, these phosphorylations are present in inactive states and are part of proper kinase folding. Complete activation of cPKCs requires binding to calcium and phospholipids (diacylglycerol and phosphatidyl serine)^7^,^8^,^9^.

Upon activation, PKCs undergo conformational changes that prevent intramolecular interactions and expose regions involved with binding to substrates and scaffold proteins^10^,^11^. A classic example is the intramolecular interaction between the pseudo-substrate (PS) site in the N-terminus of cPKCs and the catalytic domain. Upon activation PKCs bind isoenzyme specific ‘receptors for activated C kinases’ or RACKS. In an inactive enzyme the RACK binding site is also involved in an intramolecular interaction with a region named ψRACK, that is similar to a site in its corresponding RACK^11^. Both of these intramolecular interactions impair substrate and scaffold protein binding to the kinase, and are interrupted upon lipid binding and PKC activation^11^,^12^.

Recently, (2015) the reinterpretation of a previously reported crystal structure of PKCβII combined with docking of the different domains in the kinase, proposed an alternative structure for inactive PKC with the C2 domain interacting with the carboxy-terminus (V5) and the catalytic domain of the kinase further contributing to the maintenance of the kinase in an inactive conformation^10^. These findings shed light to the idea that it is possible to explore PKC´s structure for epitopes that become exposed only in an active kinase. A peptide derived from an interaction region between the C2 and catalytic domain of cPKC in an inactive kinase was chosen and used to generate active-state specific polyclonal antibodies. These antibodies preferentially recognize active cPKCs confirming that this region is exposed in the active PKC. Antibodies were then used to evaluate PKC activation in a neuroblastoma cell line, breast cancer cell lines as well as breast tumor samples from human subjects. Higher levels of active PKC was found in neuroblastoma cells upon phorbol or receptors stimulation, in triple negative breast cancer cells and in patient tissues as compared to estrogen receptor positive cells. These antibodies can be used to monitor PKC activation status and thus help elucidate the temporal dynamics of PKC activation and mechanisms that lead to tumorigenesis.

## Results

### A rational approach for the generation of PKC activation state-specific antibodies

**Figure 1:**
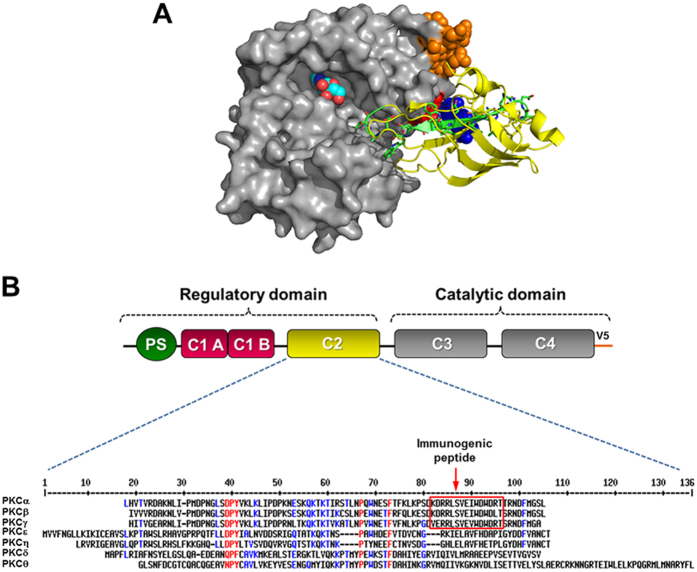
The C2Cat peptide. (**A**) Structural model of inactive PKCβΙΙ depicting intramolecular interactions between the kinase domain (grey) and the C2 domain (yellow). The V5 region also proposed to interact with the C2 domain (orange)^14^ is also shown. The C2Cat peptide localized at an interaction region between the C2 (yellow) and the catalytic domain (grey) of PKC is seen in green sticks. This region is also close to another contact region shown in space filling, K205, in the C2 domain (blue) and E655, in the catalytic domain (red)^9^. The V5 domain is also shown (orange). (**B**) Schematic representation of the primary structures of domains in classical protein kinase C (WTPKCbI) adapted from Newton, 2010^8^ and Cenni *et al.* 2002^6^. Alignment of amino acid sequence of cPKCs and nPKCs using MultAline. The C2Cat peptide (red square) used to produce anti-C2Cat antibodies is shown to be conserved in all cPKCs.

Based on the crystal structure coordinates^13^, the follow up study reinterpreting the crystal structure and validating that the C2 domain interacts with the catalytic domain in PKCβII^10^, and other biochemical studies, about the conformational changes suffered by PKC upon activation^14^,^15^, we propose a model for PKCβΙΙ similar to the one proposed by Antal and collaborators^10^ in which the kinase domain interacts with the C2 domain. This model was generated using symmetry operations searching for kinase domains in neighboring asymmetrical units that make contact with the C2 domain (Fig. 1). The criteria for selecting a peptide, epitope was, that it should be exposed only in an active kinase. A peptide named C2Cat [KDRRLSVEIWDWDLT (amino acids 236–250 of PKCβ)], (Fig. 1A, green), localized at an interaction region between the C2 (Fig. 1A, yellow) and the catalytic domain (Fig. 1A, grey) of PKC was chosen. The C2Cat peptide is mostly conserved in all cPKCs (Fig. 1C) and contains the ψRACK sequence (SVEIWD), previously proposed to interact with the RACK binding site in an inactive kinase^16^ (Fig. 1A, green). Also, this region is close to another previously described contact region [(K205, in the C2 domain and E655, in the catalytic domain (Fig. 1, blue and red, respectively)]^10^. Furthermore, the model created here includes the V5 region, also previously proposed to interact with the C2 domain^15^ (Fig. 1A, orange). The C2-kinase domain interaction and kinase domain/pseudo-substrate interaction (not shown here) would be major forces used to maintain PKCs inactive. Upon activation lipid binding disrupts these interactions exposing the contact regions only in an active kinase. Thus upon activation C2Cat would become exposed, and anti-C2Cat antibodies should specifically recognize active cPKCs.

### Anti-C2Cat antibodies preferentially recognize active cPKCs

**Figure 2:**
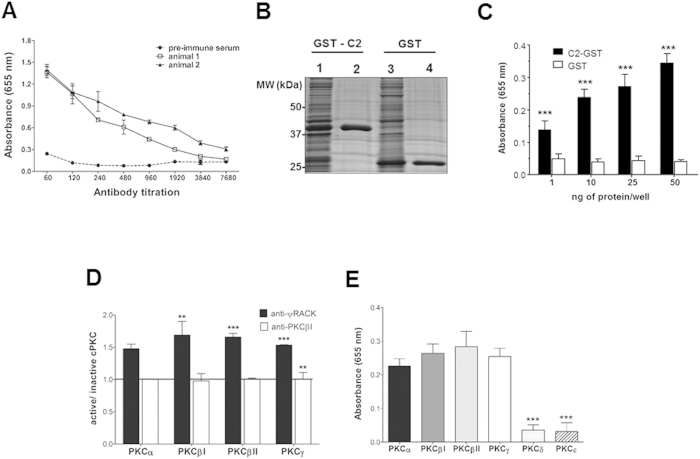
Anti-C2Cat antibodies preferentially recognize active cPKCs. (**A**) Reactivity of sera obtained from rabbits immunized with C2Cat-KLH to C2Cat-peptide coupled to BSA was determined by ELISA as described in Methods (n = 3). (**B**) SDS PAGE and Coomassie blue staining of lysates from induced bacteria expressing cPKC C2-GST (lane1), purified C2-GST (lane 2), induced bacteria expressing GST (lane 3), and purified GST (lane 4). (**C**) Recognition of cPKC C2-GST domain, and GST alone by anti-C2Cat was evaluated by ELISA assay. Anti-C2Cat was diluted 1:50 (n = 3). (**D**) ELISA assay used to detect the reactivity of anti-C2Cat to active cPKC isoenzymes α, βΙ, βΙΙ and γ (with phospholipids and calcium)/inactive cPKC (100 ng) compared to commercially available anti-PKCs: α, βΙ, βΙΙ, γ antibodies that recognizes the isoenzyme specific V5 domains (1:500) (n = 3). (**E**) ELISA assay used to detect the reactivity of anti-C2Cat to cPKCs (α, βΙ, βΙΙ, and γ) and nPKCs (δ and ε) (n = 3). Statistics was determined using ANOVA-Bonferroni test where ***p < 0.001 and **p < 0.01.

To generate activation specific polyclonal antibodies, rabbits were immunized with C2Cat peptide (Fig. 1B) coupled to KLH. Sera from two immunized rabbits recognized the C2Cat peptide up to a 1:3840 dilution (Fig. 2A). For subsequent experiments, serum from animal 2 was used as it showed a slightly higher antibody titer. Anti-C2Cat antibodies also recognized purified GST-coupled C2 domain of PKCβ but not GST alone (Fig. 2C). This antibody recognized active PKC (activated by the presence of phospholipids and calcium) 1.5 times better than unactivated cPKCs (α, βΙ, βΙΙ and γ) at 1:50 dilution (Fig. 2D). This is in contrast to commercial antibodies generated against the V5 domain of individual cPKC isoenzymes that were not able to differentially recognize active versus inactive cPKCs. As expected, the anti-C2Cat antibodies did not react with nPKCs (Fig. 2E). Together, these results indicate that anti-C2Cat antibodies selectively recognize active cPKCs with higher affinity.

**Figure 3:**
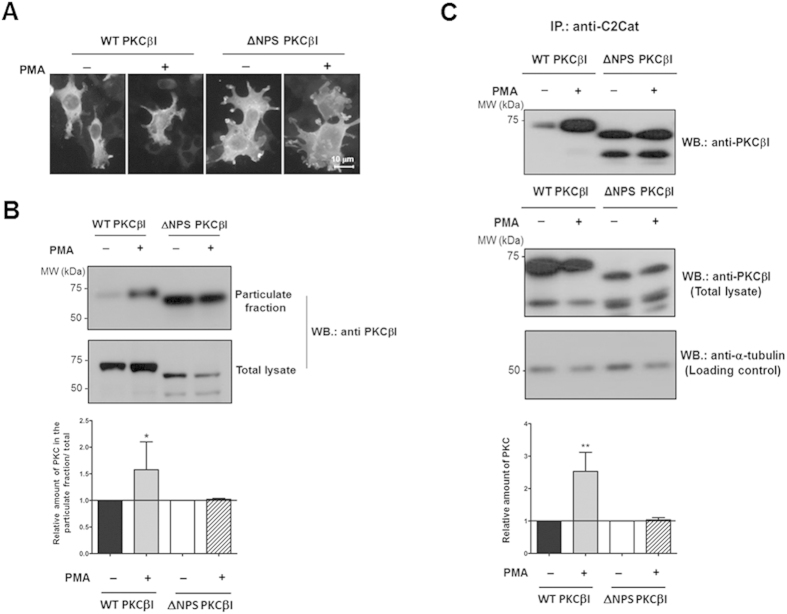
Anti-C2Cat antibodies immunoprecipitate more cPKC upon activation with PMA. (**A**) HEK293T cells were transfected with either WTPKCβΙ or ΔNPSPKCβΙ and treated with 100 nM PMA for 15 minutes. Fixed cells were then incubated with anti-PKCβΙ V5 domain antibodies and subsequently with anti-rabbit antibodies labeled with Alexa 555. (**B**) To evaluate the amount of active (membrane bound) PKCβΙ in HEK293T transfected cells. Cells treated or non-treated with 100 nM PMA were fractionated and probed for PKCβΙ in the particulate fraction by Western blot with anti-PKCβΙ-V5. (**C**) Transfected HEK293T cells were treated with 100 nM PMA and control cells were immunoprecipated with anti-C2Cat antibodies and probed for PKCβΙ with anti-PKCβΙ-V5 (upper panel). Total lysates were probed with anti-PKCβΙ to evaluate transfection levels, and for α-tubulin to evaluate the total amount of protein loaded. A representative blot of n = 3 is shown for (**B**,**C**). Quantitative analysis of the average of three independent experiments is shown normalized to non-treated cells, which was set to 1. Statistical significance was determine by ANOVA-Bonferroni test where **p < 0.01 and *p < 0.05.

Next, the ability of the anti-C2Cat antibody to immunoprecipitate and localize active PKC was evaluated. HEK293T cells were transfected with plasmids to express either wild type PKC (WTPKCβΙ) or N-terminally truncated PKC that would lead to constitutive activation (using a construct without the first 30 amino acids where the pseudo-substrate site is located, ΔNPSPKCβΙ, and thus found in a permanently active conformation). Immunofluorescence and Western blot analysis of the particulate fractions reveal that the cells expressing wild type PKC, when probed with commercial antibodies (against the V5 domain of PKCβΙ) showed more membrane bound (active) PKC upon treatment with 100 nM PMA for 15 minutes as compared to control cells (Fig. 3A,B). In contrast, cells expressing the N-terminally truncated, constitutively active PKC showed high levels of membrane bound (active PKC) in the absence of PMA treatment and the treatment did not lead to an additional increase in the levels of active PKC (Fig. 3A,B). When lysates from transfected cells that had been activated by PMA were immunoprecipitated with anti-C2Cat, an increased level of cPKC was detected from wild type PKC expressing cells that were treated with PMA, as compared to those in non-treated cells. As expected, anti-C2Cat immunoprecipitated high levels of active PKC from cells transfected with ΔNPSPKCβΙ, regardless of PMA treatment (Fig. 3C upper panel). Together these results support the ability of the anti-C2Cat antibody to selectively and consistently detect active cPKC in cells.

### Anti-C2Cat detected PMA and receptor triggered activation of cPKC in the neuroblastoma cell line SK-N-SH

**Figure 4:**
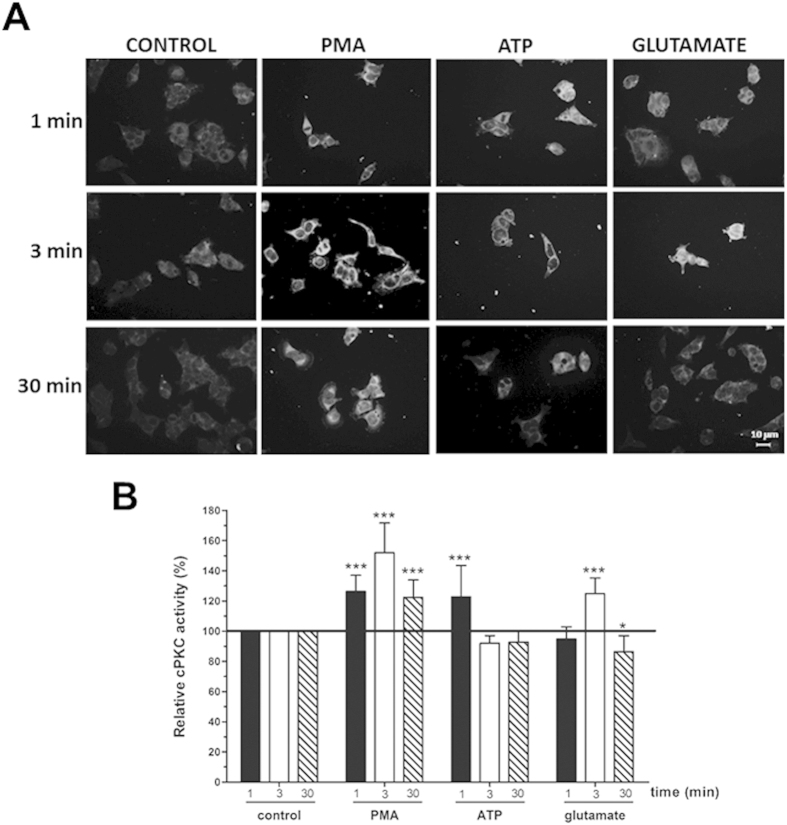
Detection of receptor and Phorbol ester triggered cPKC activation with anti-C2Cat. (**A**) SK-N-SH cells were treated with 1 μM of ATP or glutamate or 100 nM PMA for indicated periods, activators were removed and cells fixed for immunofluorescence analysis as described in Methods. Specific cPKC activation was assessed using anti-C2Cat and secondary antibody conjugated with Alexa 568. (**B**) The level of fluorescence intensity was quantified using ImageJ^®^ software and the amount of cPKC activity in each treatment was normalized to the control levels (fluorescence detected in unstimulated cells), which was set to 100. Results represent the average ±SD of measurements from twelve to fifteen different images, statistical significance was determine by ANOVA-Dunnett’s test where ***p < 0.001 and *p < 0.05.

To further explore if anti-C2Cat could be used to detect active PKC, a human neuroblastoma cell line (SK-N-SH) was stimulated with PMA or selective agonists. This cell line has been useful in the characterization of signaling pathway of a variety of receptors^17^. SK-N-SH cells were treated with either 50 nM PMA or 1 μm of ATP or glutamate for 1, 3 or 30 minutes and examined for the relative activation of cPKC using immunofluorescence with anti-C2Cat antibody. A representative figure is shown (Fig. 4). Activation of cPKC by PMA was sustained up to 30 minutes while peak activation by ATP was seen at 1 minute and glutamate at 3 minutes of treatment. These results are consistent with previous reports in other cell lines where receptor mediated activation of PKC has been found to be rapid and transient as compared to activation of PKC by PMA^11^. Together, these results show that anti-C2Cat antibody can be used to monitor spatial and temporal dynamics of cPKC activation in addition to the relative levels of active cPKC.

### Anti-C2Cat detects more active cPKCs in triple negative MDA-MB-231 compared to estrogen receptor positive MCF-7 breast cancer cell lines

**Figure 5:**
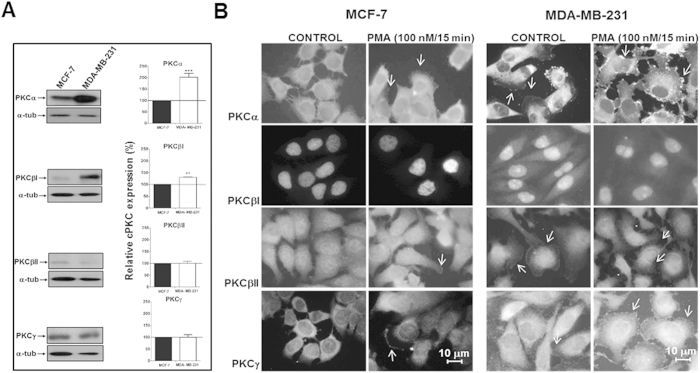
Expression and subcellular localization of cPKCs in ER+, MCF-7, and triple negative, MDA-MB-231 breast cancer cell lines. (**A**) Expression of cPKCs in MCF-7 and MDA-MB-231 was determined by Western blot using commercially available isoenzyme specific rabbit polyclonal antibodies against the V5 domain of specific cPKCs. As a loading control lysates were probed for α-tubulin. A representative blot of n = 3 is shown. Quantitative analysis of three individual experiments are shown normalized to α-tubulin and relative to expression in MCF-7 cells, set to 100. Statistical significance was determine by ANOVA-Bonferroni test where ***p < 0.001 and **p < 0.01. (**B**) Immunolocalization of cPKCs (α, βΙ, βΙΙ and γ), using commercially available isoenzyme specific mouse (PKCβΙ) and rabbit polyclonal antibodies anti V5 domains, in MCF-7 and MDA-MB-231 cells treated or non-treated with PMA (100 nM for 15 minutes). Anti-rabbit and anti-mouse Alexa 555 conjugated antibodies were used to detect reactivity of antibodies representative images. White arrows indicate PKC localized to the membrane.

Previous studies suggested that the estrogen dependent MCF-7 breast cancer cell line has lower levels of active PKC than the estrogen independent, Ras transformed, triple negative, breast cancer cell line MDA-MB-231^18^,^19^. To investigate this further, the ability of anti-C2Cat antibody to differentially detect active cPKC in these cells lines was examined. First, the expression and subcellular localization of the different cPKCs in these two cell lines was evaluated. MDA-MB-231 expressed two-fold higher levels of PKCα, slightly higher levels of PKCβΙ and similar amounts of other cPKCs (βΙΙ and γ) as compared to MCF-7 cells (Fig. 5A). Next, the subcellular localization of different cPKCs in cells treated or non-treated with 100 nM PMA, was assessed using isoenzyme specific antibodies. PKCα and γ translocation to the membrane was evident in both cell lines upon activation with PMA. PKCγ was also found in the perinuclear region upon activation with PMA. PKCβΙ was mainly localized to the nucleus with or without PMA. PKCβΙΙ translocated to nuclear and perinuclear regions and plasma membrane. Similar subcellular localizations were observed in both cell lines. The expression of PKCα as seen both by Western blot and immunofluorescence was greater in MDA-MB-231 compared to MCF-7 cells (Fig. 5B).

**Figure 6:**
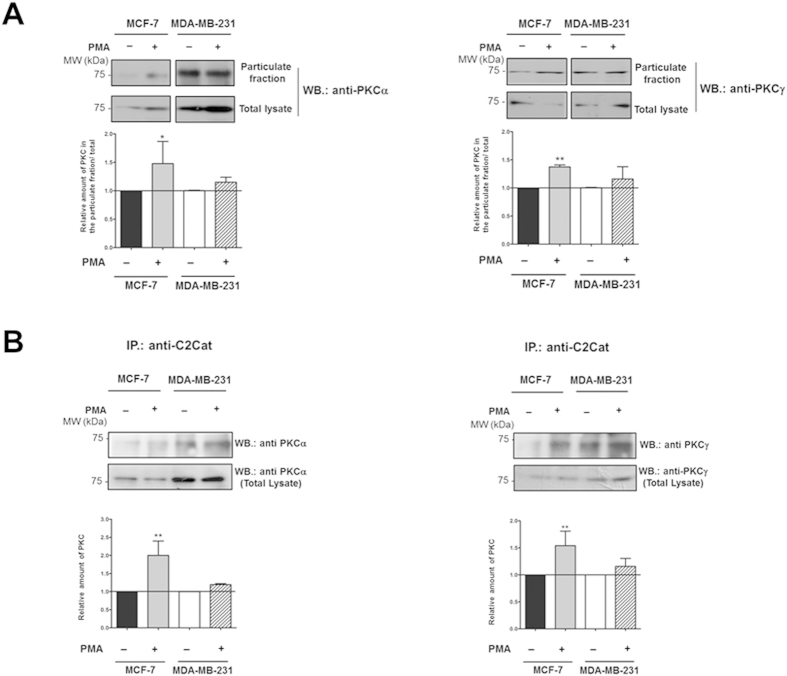
There is more active cPKC in MDA-MB231 cells as compared to MCF-7 cells as determined by reactivity with anti-C2Cat antibodies. (**A**) MDA-MB-231 and MCF-7 cells were treated or non-treated with PMA as described above in legend to Fig. 5B. Cell lysates were prepared, fractionated and analyzed by Western blot. The amount of α and γ PKC present in the particulate fraction (active PKC) relative to the total amount of kinase was determined by probing for PKCα and γ with anti-V5 domain antibodies. Quantitative analysis and statistical significance determined by ANOVA-Bonferroni test (n = 3) where **p < 0.01 and *p < 0.05 (**B**) Immunoprecipitation of active PKCα and γ, by anti-C2Cat, from MDA-MB-231 and MCF-7 cells treated or non-treated with PMA as described above. Lysates were prepared and immunoprecipitated with anti-C2Cat and probed for PKCα and γ using commercially available rabbit polyclonal antibodies against isoenzyme specific V5 domains (representative experiment of n = 3) Quantitative analysis and statistical significance determined by ANOVA-Bonferroni test (n = 3) where **p < 0.01.

It is widely accepted that membrane bound PKC correlates with active PKC (Kraft and Anderson 1983). To verify that PKCα and γ are in fact more active in MDA-MB-231 cells, and hence membrane bound, fractionation experiments were performed. Western blots of particulate fractions with commercially available antibodies anti-PKCα and γ indicated that the total amounts of PKCα and γ in the particulate fraction is higher in MDA-MB-231 cells as compared to MCF-7 cells (Fig. 6A). The amount of active PKCα and γ (PKC in the particulate fraction) in MCF-7 cells increased upon PMA treatment. This was not evident in MDA-MB-231 cells presumably due to the already high levels of active cPKCs in this cell line (Fig. 6A).

Immunoprecipitation assays with anti-C2Cat antibodies confirmed that there is more active cPKC (α and γ) in MDA-MB-231 cells (Fig. 6B). Interestingly, despite the fact that similar amounts of PKCγ are expressed in both cell lines, cPKCα and γ were immunoprecipitated by anti-C2Cat only from MDA-MB231 cells (Fig. 6B). Upon treatment with PMA anti-C2Cat was able to immunoprecipitate PKCs α and γ form MCF-7 cells and only a slight increase in the immunoprecipitation of these isoenzymes was observed from PMA treated MDA-MB-231 cells, due to its high baseline activation status (Fig. 5B).

**Figure 7:**
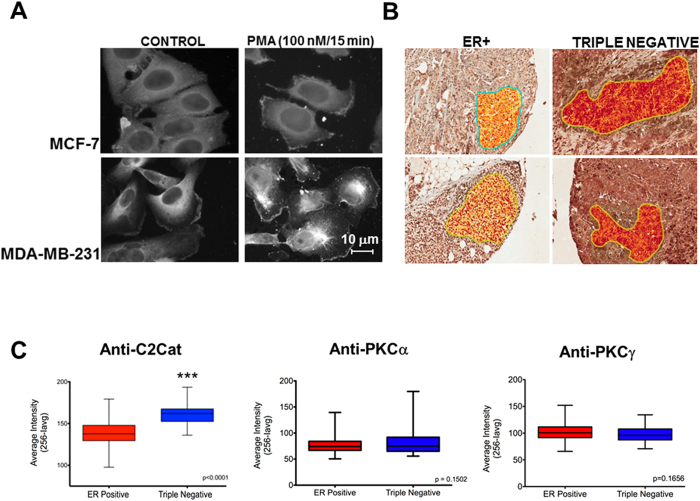
There are higher levels of active cPKC in human invasive breast cancer triple negative cells and tumors as compared to ER+. (**A**) Immunolocalization of active cPKCs as detected by anti-C2Cat antibody in cells treated or non-treated with PMA (100 nM for 15 minutes), in triple negative (MDA-MB231) cells and in ER+ (MCF-7) cells. (**B**) Representative examples of breast tumor samples from patients with ER+ and triple negative tumors probed with anti-C2Cat. An area containing exclusively tumor cells was selected by an experienced pathologist and evaluated using digital automated quantification, where red pixels indicate strong positive staining, orange positive staining and yellow weak positive staining according to the default parameter settings strong positive <220, positive = 220-175, weak positive = 175-100 and negative = 100-0). (**C**) Quantitative analysis of 105 ER+ and 29 triple negative samples probed with anti-C2Cat and commercially available anti PKCα (sc-208) and γ (SC-211) antibodies (1:100), where ***p < 0.0001, unpaired t-test. Y-axis scale represents stained tissue light absorbance and is expressed as 256-Intensity average to show a higher box-plot for triple negative tumors.

Next, we evaluated the subcellular localization of active PKC in MCF-7 and MDA-MB-231 cells using anti-C2Cat antibody. Antibody specificity was indicated by blocking C2Cat reactivity with an immunizing peptide (Supplementary figure 1).The intensity of staining was higher in MDA-MB-231 cells as compared to MCF-7 cells under basal conditions (Fig. 7A). In MDA-MB-231 cells, active PKC was found mainly at the membrane, possibly in migrating foci, and in the perinuclear region. PKCα and γ were detected at the same regions (Fig. 5). Some staining was also found in the nucleus probably due to PKC b. The finding of active PKC at these locations even in the absence of PMA corroborates that MDA-MB-231 has higher levels of active cPKC as compared to MCF-7 cells. Addition of PMA increased the signal both in the perinucleus and plasma membrane, an effect seen more clearly in MCF-7 cells (Fig. 7A). Together, by subcellular fractionation, immunoprecipitation and immunofluorescence with the activation-state specific anti-C2Cat antibodies these studies show that cPKCα and γ are more active in the triple negative MDA-MB-231 cells as compared to MCF-7 cells.

### Anti-C2Cat detects more active cPKCs in triple negative than estrogen positive tissues from human invasive breast cancer

Since phosphorylation does not correlate with activation status^7^,^8^,^9^, PKC activation has been seen by membrane translocation^4^ or FRET based phosphorylation probes^20^. However, these tools are not useful to determine cPKC activation status in tumor samples. Thus, a limitation in understanding the role of PKCs in tumorigenesis is the lack of tools to detect active PKC in tumor samples^2^. To see if anti-C2Cat antibodies can detect active PKC in tumor samples and if as the cell lines, triple negative breast tumors have more active cPKC as compared to estrogen receptor positive (ER+) tumors, tissue micro arrays of ER+ and triple negative breast cancer tumor samples were analyzed for their reactivity with anti-C2Cat. Quantification of immunohistochemistry of tissue microarrays were performed with an Aperio ScanScope XT digital scanner (Leica Biosystems), on areas containing tumor cells only, as selected by an experienced pathologist (VPA). Pixels in red indicate strong staining, orange moderate staining and yellow weak staining according to default parameter settings (Fig. 7B). Quantitative analysis of 105 ER+ samples and 29 triple negative samples showed a significant higher average staining in samples from triple negative tumors as compared to estrogen positive tumor samples (p < 0.0001, Fig. 7C). Staining and quantification of the expression of cPKCs α and γ was also determined in these samples using commercially available antibodies and did not show significant differences in PKCα and γ expression levels in ER+ and triple negative tumors. These results reveal the usefulness of the anti-C2Cat antibody in being able to quantitatively reveal the level of active PKC in human cancer tissues.

## Discussion

Kinase activation usually correlates with phosphorylation of the activation loop^5^. However, in PKC, phosphorylation does not correlate with activation and is part of the kinase folding/maturation process. This difference in activation is due to, the fact that c and nPKCs are phosphorylated at three different sites and must bind phospholipids to achieve full-activation^9^.

With the improvement of techniques to determine protein structure and the increase in accessibility of kinase structures more information is available to help understand the conformational changes undergone by a kinase upon activation. In this context, the use of structural knowledge of cPKC can help understand the kinetics of activation and allow the creation of biotechnological tools that explore the conformational changes that occur in PKC upon activation. Recent findings about how activation domains interact with the kinase domain in cPKC^10^ shed light to this field, allowing the selection of specific epitopes to design probes for desired functional forms. Certainly, this is a great advance for studies of cPKC biology. To our knowledge taking advantage of the information from these activity-mediated conformational changes is seldom used and may be a new strategy to develop kinase or even small G protein activity-state specific antibodies. These tools will be helpful not only to further understand the biology of a specific kinase but also may be applied to evaluate the activity status of proteins in different pathologies or depending on the target have therapeutic applications. Moreover, phosphorylation is labile and commonly lost during tissue handling, resulting in false negatives with anti-phospho-specific antibodies^6^ to this end an activity-state specific conformational antibody would be more advantageous.

Receptor-mediated activation of PKC has been shown to be more rapid and transient than phorbol ester-activation,^11^. Using anti-C2Cat and a neuroblastoma cell line we compared the time course of receptor and PMA mediated activation of cPKC and confirmed that PMA activation of cPKC is sustained as compared to receptor activation. Spatial, temporal and activation levels of kinases, like ERK and PI3 kinase have been shown to be important in determining cell fate^21^. Anti-C2Cat can aid in determining the role of PKC in cell fate, physiological and pathological processes even at the single cell level looking at the endogenous kinase, without the need of transfecting cells for PKC activity probes^20^.

Protein kinase C has been seen as a tumor promoter, however, PKC inhibitors have not been successful in clinical trials and recent evidences (2015) suggest that this may be due to a duality of cPKC functioning both as a tumor suppressor and promoter^1^. Further, PKC was shown to be reduced in tumors that it would have a tumor suppressor function and to contain mutations that would inactivate the kinase, and thus inhibit its suppressor function^1^. Therefore, prior to treatment with PKC inhibitors it would be important to verify not only kinase expression but also activation status.

An interesting point is that there are few mutations found in PKC in breast tumors^1^; suggesting that in this cancer PKC may not be a tumor suppressor. The precise role of PKC in breast cancer still needs to be determined. Previous studies suggested that there may be different roles for PKCs in different types of breast tumors and there is an overall increase in PKC activity in triple negative cell lines as compared to ER^+^ cells^22^. Using anti-C2Cat antibodies an increased activity of cPKCs was detected in triple negative cells and tumor samples from humans. PKCα expression levels were more variable than PKCγ levels in the tumor samples, and there was a slight tendency for an increase in PKCα expression levels in triple negative tumors. However, differences in expression levels of cPKCs α and γ in the same triple negative tumors as compared to ER^+^ tumors stained with anti C2Cat were not significant suggesting that rather activation status and not expression levels may account for different PKC mediated signaling pathways in these tumors.

Taken together, a new strategy to probe for active cPKC was developed and validated in different models. This tool can be used to detect active PKC and may be helpful to establish the cause-effect of PKC on cancer and guide therapeutic strategies. Further, since conformational changes upon activation are a common theme in signal transduction, the same strategy can be used to develop antibodies for other signaling proteins.

## Materials and Methods

### Materials

Antibodies against the V5 domain of PKCα (sc-208), PKCβI (sc-8049 and sc-209), PKCβII (sc-210) and PKCγ (SC-211) were purchased from Santa Cruz Biotechnology^®^. Anti-α-tubulin antibody (T9026) was purchased from Sigma-Aldrich^®^. Recombinant proteins PKCα, PKCβI, PKCβII, PKCγ, PKCδ and PKCε produced in Sf9 cells were obtained from Invitrogen^®^. Phosphatidylserine (PS) and 1-2-dioleoyl-sn-glycerol (DG) were purchased from Avanti Polar Lipids^®^ Phorbol myristate acetate (PMA) was obtained from Sigma-Aldrich^®^. TMB substrate (3,3′, 5,5′ tetramethylbenzidine) was purchased from BD biosciences^®^ and secondary antibodies conjugated with Alexa 555 and Alexa 568 were purchased from Molecular Probes^®^.

### Generation of a PKCβ model of inactive kinase

Using YASARA software^23^ based on the crystal structure coordinates for PKCβΙΙ PDB 3PFQ^13^, we used symmetry operations to search for kinase domains in neighboring asymmetrical units that make contact with the C2 domain. We deleted the residues forming the long connector between C1A and C2 domains and the original C2 from the asymmetric unit deposited in the PDB. A new position for the C2 domain was docked on the kinase domain, using space group symmetry operations. We confirmed that in our model there was the interaction between K205, in the C2 domain and E655, in the catalytic domain described and validates by Antal and collaborators^10^.

### Cell Culture

Human Embryonic Kidney (HEK293T) cells were obtained from Dr. Malnic B. (Universidade de São Paulo, Instituto de Química) and Neuroblastoma cells (SK-N-SH) were obtained from American Type Culture Collection (Manassas, VA, USA). Both cells lines were maintained in DMEM high Glucose supplemented with 10% fetal bovine serum (FBS), penicillin/streptomycin [50 U/ml and 50 μg/ml respectively (Gibco-BRL^®^)] at 37 °C under 5% CO_2_.

Human MCF-7 and MDA-MB-231 breast cancer cell lines were obtained from Dr. Labriola L. (Universidade de São Paulo, Instituto de Química) and maintained in DMEM/F12 and RPMI-1640 (Sigma-Aldrich^®^), respectively, both media were phenol red free and supplemented with penicillin/streptomycin and 10% FBS in a humidified atmosphere at 37 °C and 5% CO_2_.

### Plasmids and constructs

The C2 domain of PKCβI (amino acids 152–292) was subcloned in pGEX 4T-1 vector (kindly provided by Dr. Gomes, S.L., Universidade de São Paulo, Instituto de Química) between the restriction enzyme sites *Bam* HI and *Xho* I using the following primers: forward 5′ ATTGATGGATCCTACATGCAGGCCCACATC 3′; reverse 5´ ATACATCTCGAGGCCTTCTTCCTGGCTTAG 3′, this region was amplified from cDNA for PKCβI obtained from PKCLab.org. The C2 domain was expressed as a fusion protein with GST (glutathione S-transferase) in *Esherichia coli* BL21 (DE3). C2-GST and GST proteins were purified by affinity chromatography on glutathione resin according to the GE Healthcare protocol. Constructions expressing the Wild type PKCβI (Wt PKCβI) and PKCβI containing a deletion of the first thirty amino acids of the N-terminus, including the pseudo-substrate (∆NPS PKCβI) were both cloned in the mammalian expression vector pcDNA 3.1 in *Bam* HI and *Xho* I sites using the following primers: forward 5′ ATATTTAGGATCCCAGATGGCTGACCCG 3′/reverse 5´ CAGGTCGGTCTCGAGCTACACATTAATGAC 3′ and forward 5′ A TATTTAGGATCCCAGATGAACGTGCACGAG 3′ reverse 5′ CAGGTCGGTCTCGAGCTACACATTAATGAC 3′, respectively.

### Transfection of HEK293T cells

Transfections were performed using 25.6 μL of polyethylenimine polymer (PEI) (1 mg/mL) diluted in 520 μL of serum-free DMEM high glucose, 8 μg of DNA was added into the solution and incubated for 10 minutes. Subsequently, the DNA/PEI mix was added to the plate containing cells in DMEM high glucose supplemented with 10% FBS without antibiotics at 60–80% confluence. Transfected cells were maintained at 37 °C with 5% CO_2_ for 24 hours. Cells were then either lysed for immunoprecipitation assays or fixed with 4% paraformaldehyde and stained with anti-PKCβI, at a final concentration of 2 μg/mL as described below. Fluorescence was detected using a Nikon Eclipse E600 microscope.

### Subcellular fractionation of cells

HEK293T, MCF-7 and MDA-MB-231 cells at 80% confluence were lysed in homogenization buffer (20 mM Tris-Cl buffer pH 7.4, containing 2 mM EDTA, 10 mM EGTA, 250 mM sucrose), containing protease inhibitors diluted 1:10 (Sigma-Aldrich^®^) and phosphatase inhibitor cocktail PhosStop diluted 1:10 (Roche^®^). Cells were then fractionated into soluble and particulate fractions. Cells resuspended in homogenization buffer were ultracentrifuged at 100,000 g for 40 minutes and the supernatant collected. The pellet (particulate fraction) was resuspended in DE buffer (20 mM Tris-HCl, pH 7.5, 1 mM EDTA, 1 mM EGTA, 1% Triton-X 100 with protease and phosphates inhibitors as above.

### Generation of polyclonal anti-C2Cat antibodies

The C2Cat peptide (KDRRLSVEIWDWDLT) used for immunization was obtained from (Proteimax^®^). Glutaraldehyde conjugation of the peptide to KLH (Sigma-Aldrich^®^) or BSA was performed according to the manufacturer. Coupled peptides were dialyzed against PBS for 24 hours at 4 °C. Immunization experiments were performed with two rabbits acquired from our animal facility (Biotério de Produção e Experimentação da Faculdade de Ciências Farmacêuticas e do Instituto de Química da USP). All procedures followed specifications of the Guide for Care and Use of Laboratory Animals, National Press, USA. Emulsion of the C2Cat peptide coupled to KLH (500 μg) and Complete Freund’s Adjuvant was administered intramuscularly. Four booster immunizations at 2 weeks intervals were performed with the emulsion prepared using Incomplete Freund’s Adjuvant. Pre and post-immunization blood samples were collected and antibody titer determined by ELISA.

### ELISA assay

C2Cat peptide (100 μg) coupled to BSA, purified cPKC C2-GST or GST (1,10, 25, and 50 ng) or purified PKCβI (100 ng), diluted in PBS were incubated in 96-well ELISA plates overnight at 4 °C. Plates were washed three times with PBS/0.05% Tween-20 and blocked with 5% non-fat milk in PBS for one hour at 37 °C. Next, 100 μL of serial serum dilutions (1:120–1:3840) were added, and plates incubated for 2 h at 37 °C, and washed as above. 100 μL of HRP-goat anti-mouse IgG (Sigma-Aldrich^®^) was subsequently added and plates incubated for one hour at 37 °C, and washed. TMB substrate (BD biosciences^®^) was added and absorbance read at 655 nm using Biotek Synergy HT Multi-Mode Microplate Reader.

Assays that determined the recognition of active cPKCs by ELISA, were performed as previously described^24^. Briefly, 100 ng recombinant PKC was incubated with 40 μM ATP, 40 mM MgCl2, 2 mM CaCl2, 60 μg/mL phosphatidylserine (PS), 2 μg/mL dioleoylglycerol (DG) in Tris-HCl 20 mM pH 7.4 at 37 °C for 30 minutes. The mixture was diluted 100 times in 20 mM Tris in 96-well ELISA plates and incubated for 1 hour at room temperature. Assays were performed as described above. The affinity for active cPKC was determined by the absorbance ratio in the presence/absence of PKC activators (lipids and Ca^2+^).

### Immunoprecipitation Assays

Anti-C2Cat (5 μL) was incubated with 50 μL of packed volume of protein G (Invitrogen^®^) for 2 h at 4 °C. Antibody-bound beads were washed twice with PBS and blocked with 1% BSA for 1 h at 4 °C. Transfected cells were lysed in PBS/1% Triton X-100, containing protease (Sigma-Aldrich^®^), and phosphatase [PhosStop (Roche^®^)] inhibitor cocktails, followed by three freeze thaw cycles. Cells were sonicated for 30 minutes at 80 Hz (output) with a probe sonicator (Branson Sonifier 250). Cell lysates were precleared with protein G beads for 1 h at 4 °C, incubated with antibody-bound beads overnight at 4 °C, and subsequently washed with PBS. Laemmli buffer was added to immunoprecipitated proteins, which were analyzed by Western blot with specific PKC isoenzyme antibodies.

### Immunofluorescence

MDA-MB-231 and MCF-7 cells were cultured on 13 mm glass cover slips at 80% confluence and fixed with 4% PFA, permeabilized with PBS 0.1% Triton X-100 and blocked in PBS, 0.1% Triton-X100, 1% normal goat serum, for 40 minutes at room temperature. Cells were subsequently incubated overnight at 4 °C with anti-PKC specific isoenzyme antibodies, (2 μg/mL) in blocking solution or with anti-C2Cat serum diluted 1:100, and incubated for 1 hour at room temperature with anti- rabbit conjugated to Alexa 555 (4 μg/mL) diluted as above. Coverslips were mounted with Vectashield^®^/DAPI. Immunofluorescence staining was analyzed using a Nikon Eclipse E600 or with a Leica DM6000 fluorescent microscopes. For immunofluorescence using SK-N-SH, cells were plated at 30% confluence and treated with 50 nM PMA or 1 μM of each ligands (ATP or glutamate) for 1, 3 and 30 minutes. After treatment, cells were fixed with 4% PFA and immunofluorescence performed as described above.

### Tissue Micro Array immunohistochemistry (IHC)

Study approval for analyses of human samples was obtained from the AC Camargo Cancer Center Ethical Committee (process #08284/2015). All patients signed a general informed consent before tumor tissue removal allowing the use of their tissue for research purposes. The study was carried out in accordance with the approved guidelines and regulations. Files of human breast cancer samples from the Department of Pathology at AC Camargo Cancer Center, Sao Paulo, Brazil were reviewed and representative areas from each tumor were selected for construction of two recipient Tissue Microarray blocks, one with ER+ samples and the other with ER-/PR-/HER2- samples. Donor blocks were punched using 1 mm needles using the Manual Tissue Arrayer MTA-1 (Becher Instruments^®^) and inserted at 0.2 mm intervals. A total of 105 ER+ and 29 triple negative tumors were evaluated after IHC staining and digital scanning.

Slides containing 1mm diameter tissue samples were cut at 4 m thickness and stained in Benchmark ULTRA (Roche-Ventana^®^) immunohistochemistry autostainer after anti-C2Cat dilution. Deparafinization was performed in EZ PREP and antigen retrieval by heat in Ultra Cell Conditionig Solution at a high pH at 96 °C for 36 minutes. Endogenous peroxidase was blocked for five minutes using the peroxidase blocking agent (UltraView Universal DAB Inhibitor diluted in 3% H_2_O_2_), followed by washing in PBS. Slides were then incubated for 32 minutes with anti-C2Cat diluted 1/300. The reaction was developed with HRP polymer (HRP Multimer) washed with PBS, incubated with Diaminobenzidine (DAB), washed in PBS and counterstained with Hematoxilin (Hematoxylin II (Roche-Ventana). Slides were mounted on Tissue-Tek film (Sakura). Quantification of immunohistochemistry was performed using the Aperio ScanScope XT (Leica Biosystems^®^) digital scanner using the PixelCount v9.0 algorithm per manufacturer´s instructions^25^.

## Additional Information

**How to cite this article**: Pena, D. A. *et al.* Rational design and validation of an anti-protein kinase C active-state specific antibody based on conformational changes. *Sci. Rep.* **6**, 22114; doi: 10.1038/srep22114 (2016).

## Supplementary Material

Supplementary Information
