# Supplementary material for: Rational design and validation of an anti-protein kinase C active-state specific antibody based on conformational changes
Source: Sci Rep. 2016 Feb 25;6:22114. doi: 10.1038/srep22114 (PMC4766434; doi:10.1038/srep22114)
Supplement: Supplementary Information [file srep22114-s1.pdf]

Rational design and validation of an anti-protein kinase C active-state specific antibody based on conformational changes

Darlene Aparecida Pena1, Victor Piana de Andrade2, Gabriela Ávila Fernandes Silva1, José Ivanildo Neves2, Paulo Sergio Lopes de Oliveira3, Maria Julia Manso Alves1, Lakshmi A. Devi4 and Deborah Schechtman1*

Figure S1: Specificity of anti-C2Cat. Specificity of anti-C2Cat was tested by blocking the antibodies with 10 μM C2Cat peptide prior to incubation with MCF-7 and MDA-MB-231 cells. On the left fixed cells were incubated with anti-C2Cat and on the right with previously blocked antibody. Cells were then incubated with secondary anti-Rabbit labeled with Alexa 555. Nuclei were labeled with DAPI.

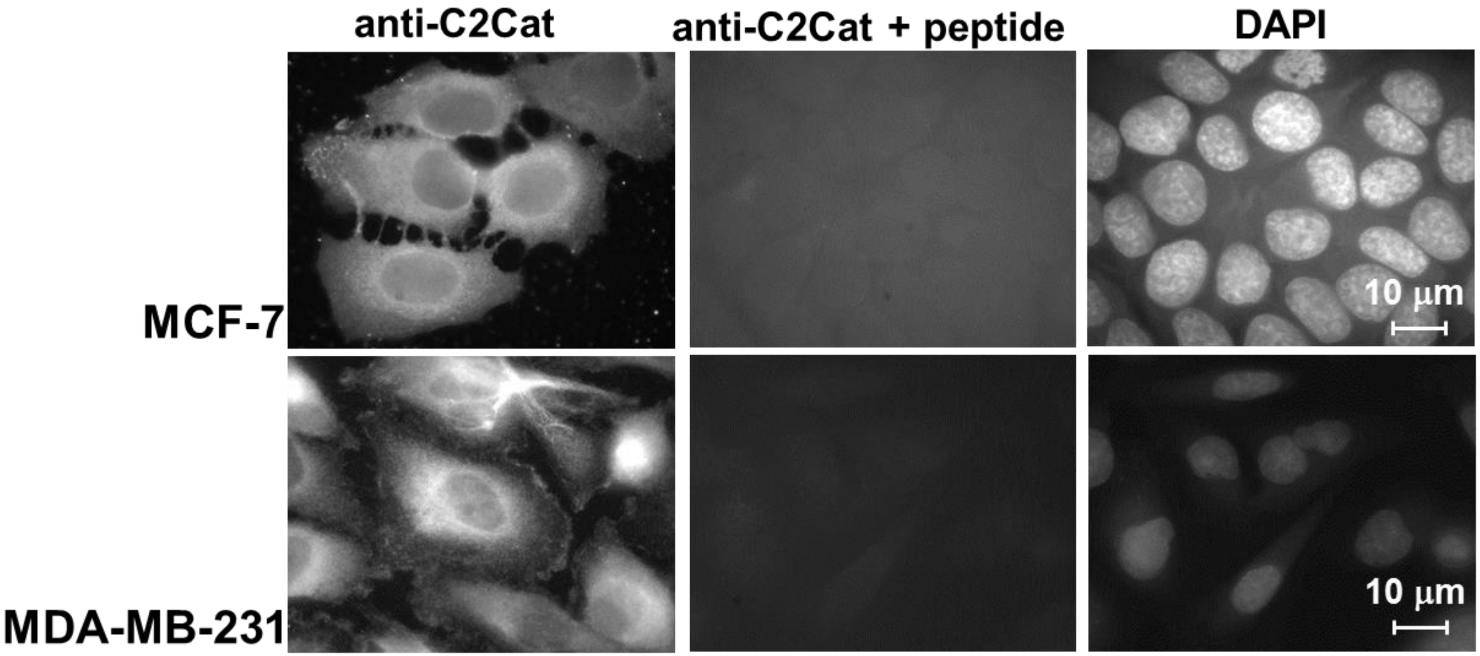
anti-C2Cat
anti-C2Cat + peptide
DAPI
MCF-7
MDA-MB-231
10 μm
10 μm
